# Morphological and genetic diversity of traditional varieties of agave in Hidalgo State, Mexico

**DOI:** 10.1371/journal.pone.0254376

**Published:** 2021-07-09

**Authors:** Carmen Julia Figueredo-Urbina, Gonzalo D. Álvarez-Ríos, Mario Adolfo García-Montes, Pablo Octavio-Aguilar

**Affiliations:** 1 Cátedras CONACYT-Laboratorio de Genética, Área Académica de Biología, Instituto de Ciencias Básicas e Ingeniería, Universidad Autónoma del Estado de Hidalgo, Mineral de la Reforma, Hidalgo, México; 2 Instituto de Investigaciones en Ecosistemas y Sustentabilidad, Universidad Nacional Autónoma de México, Morelia, Michoacán, México; 3 Laboratorio de Genética, Área Académica de Biología, Instituto de Ciencias Básicas e Ingeniería, Universidad Autónoma del Estado de Hidalgo, Mineral de la Reforma, Hidalgo, México; National Cheng Kung University, TAIWAN

## Abstract

The agaves are plants of cultural importance which have been used by humans for about 10,000 years and about 40 specific uses. The most culturally and economically important of those uses are for the production of fermented (pulque) and distilled beverages (mescal). Pulque continues to be produced in nearly all of Mexico, and the agaves used for this purpose have shown domestication syndrome. We carry out an ethnobotanical, morphological, and genetic analysis of the traditional varieties of pulque agave used in the production of *aguamiel* (agave sap) and pulque in the state of Hidalgo. We did semi-structured interviews, free listings, and tours with 11 agave managers. We analyzed morphology and studied genetic diversity and structure using nuclear microsatellites. We found wild-collected, tolerated, transplanted, and cultivated varieties of agave. This comprised 19 traditional varieties of pulque agave, 12 of them in production during the study, which corresponded to the species *Agave americana*, *A*. *salmiana* y *A*. *mapisaga* and five intraspecific entities. The varieties were grouped morphologically according to a management gradient; the wild-collected varieties were the smallest, with more lateral teeth and a larger terminal spine. The cultivated varieties clearly exhibited domestication syndrome, with larger plants and smaller dentition. The expected heterozygosity (He) of the varieties ranged from 0.204 to 0.721. Bayesian clustering suggested the existence of three genetic groups, both at the level of traditional varieties of pulque agaves and for management categories, a result that matches multivariate clustering. Pulque producers in the studied localities maintain high agrobiodiversity. The cultivated varieties exhibit domestication syndrome, as has been reported for other species of the genus with the same selection purposes. Our results support the hypothesis of a decrease in genetic diversity in crops compared to wild-growing agaves, which seems to be due to vegetative propagation, among other factors.

## Introduction

Agaves are a group of monocotyledonous, rosetophilic, succulent and monocarpic plants. They were reclassified to the Asparagaceae family, but the Agavoideae subfamily continues to be recognized. The *Agave* genus is the richest taxon within this subfamily, with 210 species distributed from the southern United States to Colombia and Venezuela and the Caribbean Islands [1–3]. In Mexico there are 160 species (76% of the genus), which can be found in various ecosystems, mainly arid and semi-arid areas, and in anthropogenic environments. Agaves are a pillar for the functioning of the systems where they grow due to several ecological functions such as soil retention-formation, erosion reduction, increased water infiltration, habitat, and food provision for species of insects, reptiles, birds and bats, as well as benefits for human populations [3,4].

According to the morphology of their inflorescences, Gentry [5] classified the genus into two subgenera: *Agave* and *Littaea*. The greatest diversity of *Agave* is found in the Tehuacan-Cuicatlan Valley, shared between the states of Oaxaca and Puebla (approximately 20 species), while the area with the greatest diversity of *Littaea* (eight species) is in the *Barranca de Metztitlán*, in the state of Hidalgo [6,7].

In the Hidalgo State, 17 *Agave* taxa have been reported, including intraspecific varieties [5]. Of these 17 taxa, four species—*Agave americana*, *A*. *lechuguilla*, *A*. *mapisaga¸* and *A*. *salmiana*—have been widely used for several purposes in the region. For example, *A*. *lechuguilla* is used to obtain fibers, make articles for cleaning and for bags. The other three species are mainly used for culinary purposes; for example, flowers are collected to prepare different dishes (*gualumbos*); the leaves (*penca*) and cuticle (*mixiote*) are used as utensils and ingredients for cooking food; drinks are prepared from the sap extracted from the central corm, both fresh as *aguamiel* and fermented as *pulque*; and preparing syrup sap (*jarabe de aguamiel*).

Archaeological records suggest that the use of agaves dates back at least 9,000 years, evidenced by findings of remains of chewed agave in different sedimentary floors in caves of the Tehuacan-Cuicatlan Valley [8–10]. There is also strong archaeological evidence of agave use in Hidalgo State; the oldest lithic records associated with the extraction of sap from agaves are found in the archeological zones of Tula and Tulancingo and are dated at 2,300 years old [11,12].

The ancient and constant interaction between humans and agaves has generated different evolutionary pressures on the plants, such as artificial selection by several cultural groups that have selected plants with characteristics to satisfy their necessities. This type of selection has exacerbated or reduced the frequency of certain characteristics of the plants, which favor the abundance or reproduction of individuals with these attributes. Plants under the same artificial selective pressures converge on a set of traits that differentiate managed individuals from their ancestors and/or wild relatives, a process known as domestication syndrome [13–16].

It has been suggested that for the agave species whose main use is the production of fresh or fermented beverages, the domestication syndrome is aimed at obtaining a greater quantity and quality of sap, which leads to plants presenting: 1) gigantism—taller overall height and leaf length and width—since larger individuals produce greater volume of sap, 2) sap with desirable organoleptic properties like higher sugar content, neutral pH, reduction in irritating compounds and structures such as saponins and raphides, and 3) reduced “thorniness—size, number, and closeness of lateral teeth and size of the terminal spine, all relative to leaf length—to avoid injury to sap collectors from these plant defense mechanisms [16–21]. This means that the terminal spine and lateral teeth are suppressed, smaller or in lesser number in comparison with the wild individuals free of selective pressures; this facilitates the manipulation of the individuals and avoids injuries of the collectors with the plants’ defense mechanisms [16,19–22].

The morphological variation and domestication syndromes of agaves used for pulque production have been studied by several authors. The results indicate that *Agave mapisaga* is the largest species and has the smallest lateral teeth; its varieties have only been recorded in anthropic environments, with no records in natural ecosystems. *A*. *salmiana* is a species with high morphological diversity, several varied of forms to the exploitation and had a gradient of domestication. On the other hand, *A*. *macroculmis* and some wild varieties such as *A*. *salmiana* ssp. *crassispina*, do not show characteristics associated with domestication syndrome [21–26].

The levels of genetic diversity of the agaves used for pulque production has only been addressed in five studies. The first was by Alfaro-Rojas and collaborators [27], who studied six varieties of cultivated agaves from the Mexican highlands using RAPDs as a molecular marker; they found low genetic diversity (He = 0.038–0.121), strong genetic structure (G_ST_ = 0.68), and low gene flow (Nm = 0.24). The second study was of the wild relative of the pulque agave, *A*. *salmiana* ssp. *crassispina* in San Luis Potosí. In that study, AFLPs were used as the molecular marker, and the authors found high genetic diversity (He = 0.403), no population structure (F_ST_ = 0) and high gene flow (tending toward infinity) [28]. The third and fourth studies were of the *A*. *hookeri* species in the *P´urhepecha* highlands in Michoacán, Mexico; nuclear microsatellites showed low population genetic diversity (He = 0.485) compared to the most likely wild ancestor (*A*. *inaequidens* He = 0.704–0.733), strong population structure (F_ST_ = 0.28) and fixation of heterozygotes, a genetic trait associated with the selection of favorable genotypes in crops [16,20]. Finally, Álvarez-Ríos et al. [21], studied five cultivated varieties in Michoacán; they also used nuclear microsatellites and found moderately high genetic diversity values (He = 0.295–0.583) and heterozygote fixation. As has occurred with agave species used for other purposes such as tequila production, cultivation of a limited number of individuals can produce a founder effect, which leads to a loss of genetic diversity. Similarly, the artificial selection of a unique genotype can also lead to a decrease of diversity and an increase in genetic structure, especially when paired with vegetative propagation. Another possible scenario is that the artificial selection of certain specific genotypes in the case of the crops come from multiple sources, in addition, there can be gene flow between cultivated genotypes and neighboring population of wild plants via pollen [20,21,29].

Hidalgo is the state with the largest cultivable area of pulque agave in Mexico. This area of approximately 4,905 ha represents 60% of the cultivation in the country for the year 2019, followed by Mexico State with 19% and Puebla with 12%. Hidalgo is also the top nationwide producer of pulque, producing 117,432,130 liters per year (68% of the annual national production) [30]. In Hidalgo, as in the rest of the country, the productive systems are made up of several species and subspecies, as well as traditional varieties of agave. However, there is no clear and precise quantification of the proportion of these sources, nor of their attributes, and there is often not even certainty as to the taxonomic identity of the plants. *Agave salmiana* var. *salmiana* and *Agave mapisaga* are the most widely used species for the extraction of sap, both in extensive crops and within the plots of small producers [21,23].

“Traditional varieties” refers to useful plants that are recognized, named, managed, propagated, and preserved by the producers [19,31,32]. These varieties are strongly associated with the knowledge of the particular producers, uses and purposes, and their formal taxonomic identity is often unclear, since some are considered intraspecific categories, or are probably of hybrid origin; this seems to be common among agaves in general [5,33,34], and has been reported specifically for traditional agave varieties from Michoacán by Álvarez-Ríos et al [21]. In the case of agaves, traditional varieties have the following characteristics: 1) they have one or more common names, sometimes in the native language of the locality, referring to obvious characteristics of the plant or to its place of origin, 2) they have been managed by humans over two or more generations of humans, 3) they are used by rural communities for multiple purposes, 4) they can cultivated or wild plants managed *in situ* or cultivated, 5) they are found to a greater extent in traditional production systems, although they can also be found in intensified systems, 6) there is a wealth of traditional knowledge and techniques associated with the management and use of each variety. These traditional varieties are of cultural and economic importance, are part of the identity of cultural groups and satisfy multiple needs—especially the nutritional needs—since the derived products are used both for self-consumption and for commercialization, generating monetary income for the families.

In localities of the Mezquital Valley, Hidalgo, 25 traditional varieties of agaves are reported. Richness is higher in indigenous than mestizo communities, but in both cases, agaves are pillars of food self-sufficiency and family economy [4,12,23,35,36].

Analyzing the characteristics and status of these plant genetic resources is essential to ensure the maintenance of the biological and cultural diversity of Hidalgo and Mexico, generate information that allows decision-making and the design of strategies for sustainable resource management, and improve the livelihood that communities will be able to maintain, manage, and continue to use these resources.

This research was carried out in two localities in the state of Hidalgo with a tradition of using agaves for producing pulque and other purposes. Our objectives were: 1) to carry out an ethnobotanical characterization of the traditional agave varieties, determine their management categories and describe the productive system, 2) to evaluate the morphological characteristics of the different varieties and to analyze their correspondence with domestication syndrome, and 3) to quantify the genetic variability of the different species and traditional varieties of agave. This will allow us to discern the consequences of management and the degree of domestication of the traditional varieties of agave whose main uses are sap extraction and pulque production in this region of Mexico. We hypothesize that agave varieties with a more intense degree of management will have morphological characteristics associated with the domestication syndrome that have been previously described for pulque agaves, while varieties that are less intensely managed will present the characteristics of the syndrome to a lesser degree. On the other hand, we expected cultivated plants to have lower genetic diversity and stronger population structure than wild-growing plants.

## Materials and methods

### Ethics approval and consent to participate

The institutions to which the authors belong do not have internal ethics committees that endorse the ethnobiological methodology applied in this research, however it has been reviewed and approved by peers of our institutions, in addition this research was carried out following the statutes of the Code of Ethics for research, action research, and ethno-scientific collaboration in Latin America of the Latin American Society of Ethnobiology (SOLAE). At the beginning of the research, we established contact with civil authorities and people in the communities and owners of agave crops. We presented the study project, its aims, and methods and asked their consent to collaborate with us. All information obtained (written testimonies, audio recordings, photographs, plant measurements in the field, and tissue samples of agaves for the genetic study) were acquired with previous express permission from the participants.

### Study area

**Fig 1:**
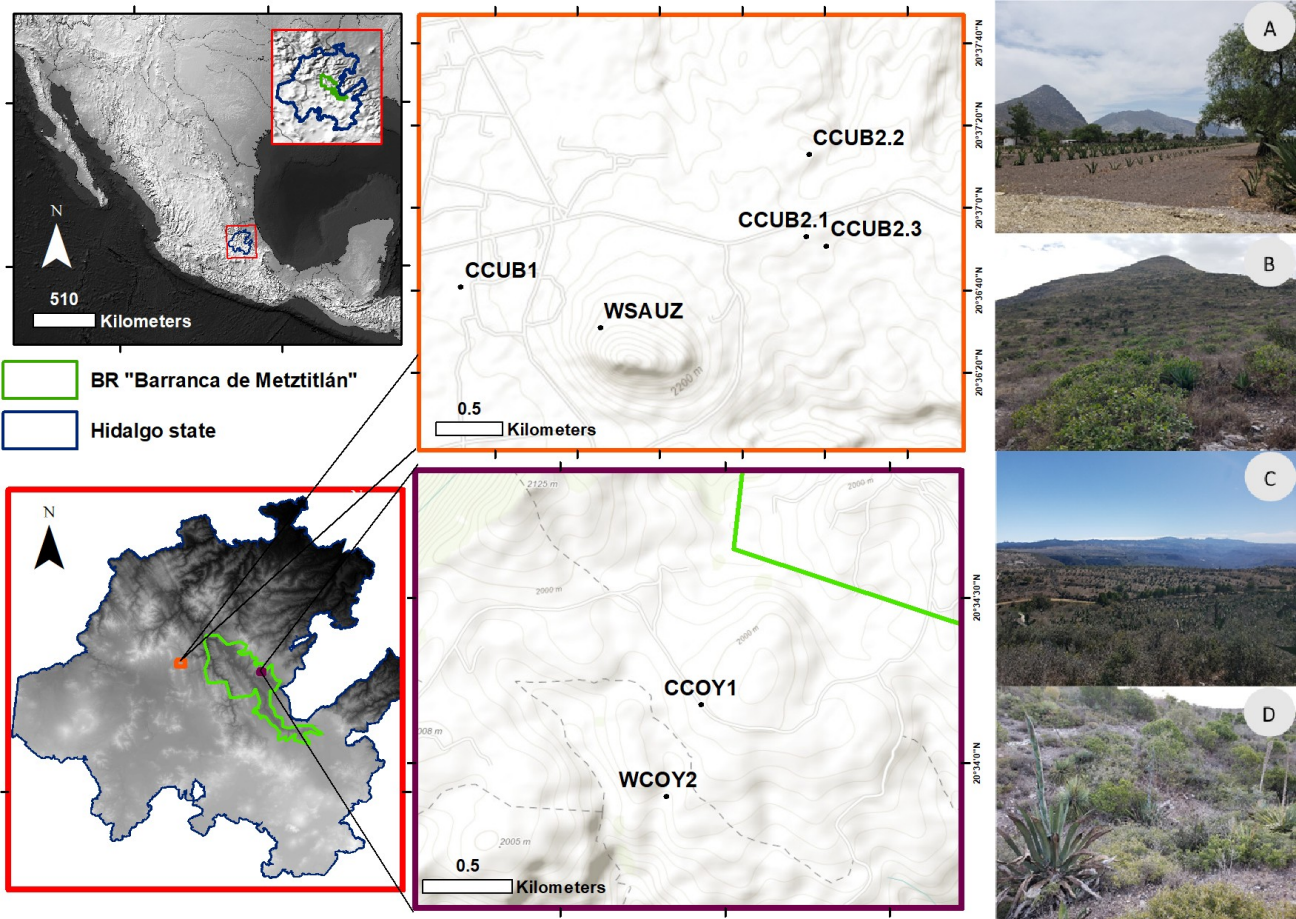
Localities studied in the state of Hidalgo (data set https://www.naturalearthdata.com/ and https://www.inegi.org.mx/app/geo2/ntm/). A) View of agave crops (Traditional varieties *Poblano*, *Agave salmiana* var. *salmiana*) as living fences and in rows interspersed with other crops (CCUB), B) Cerro Blanco or El Fraile, where agaves were measured (Traditional varieties *Corriente*, *A*. *salmiana* ssp. *crassispina*, WSAUZ), C) Panoramic view of the crops in *Jilas* in *La Coyotera* (mainly *Manso de zoqui*, *A*. *salmiana* var. *salmiana* CCOY1), D) Xeric scrubland at *La Coyotera* in the Barranca de Metztitlán Biosphere Reserve, where individuals of *Agave salmiana* ssp. *crassispina* can be seen (WCOY2).

The state of Hidalgo, located in central Mexico, has an area of 20,905 km^2^, which represents 1.1% of the total area of Mexico [37,38] and is divided into 84 municipalities. Four major physiographic features converge within the state: the Mexican Altiplano, the Trans-Mexican Volcanic Belt, the Sierra Madre Oriental and the North-eastern Coastal Altiplano [37]. This leads to a wide diversity of vegetation types. The study was carried out in two localities in Hidalgo (Fig 1). The first, *El Cubo*, in the Cardonal municipality of the Mezquital valley, has 63 inhabitants, ten of which speak the Hñähñu indigenous language [35]. Its inhabitants practice seasonal agricultural and sell products to tourists who visit the *Grutas de Tolantongo* caves, located 11 km away. There, we studied the agave plantations of two producers (CCUB1 and CCUB2, Fig 1A) and measured wild-growing agaves in *Cerro Blanco-El Fraile*, in the *El Sauz* locality, located in the same municipality (WSAUZ, Fig 1B). The second locality was the *Rancho La Coyotera*, located in the *Rincón Grande* municipality of *Zacualtipán de Ángeles* within the Barranca de Metztitlán Biosphere Reserve. It has a mestizo population of 31 people, who grow seasonal crops and raise livestock, especially goats [35]. We studied cultivated agaves in a 40 ha area (CCOY1, Fig 1C) and wild-griwing agaves found in xeric scrubland of the same locality (WCOY2) (Fig 1D).

### Ethnobotanical assessment

We collected data between February 2019 and February 2020. In order to initially contact agave managers, we asked pulque vendors from each locality who the pulque producers were in the region, who we then sought out within the community to present the project. Once we made contact with these managers, we used snowball sampling to identify and contact other agave managers in the community. We contacted eleven agave managers—six in *El Cubo* and five in *Rancho La Coyotera*. We carried out free listings, recording the traditional varieties of agaves they recognized and used. We also did a semi-structured interview (S1 File) to obtain the description of the features of each traditional variety and the management practices associated with each production system. In addition, with the agave producers who agreed to participate further in this research, we did ethnobotanical walks with the producers within their plots to recognize the traditional varieties and obtain an *in situ* record of the practices. Producers often feel more comfortable and confident when immersed in their production systems, leading to a more detailed description of the system’s particularities. We used the keys of Gentry [5] for the taxonomic identification of the traditional varieties.

### Analysis of morphometric variation of agaves

We selected agaves for morphometric measurements while accompanied by the producers. In total, 111 individuals were measured (details of the sample sizes are in Tables 2 and 5). We chose "mature" individuals (i.e., plants that would soon develop the floral scape), which is the stage at which producers remove the central meristem to collect the sap. The same selection criteria were used for wild-growing and cultivated agaves. We measured 12 morphological traits *in situ*. We measured the leaf in the third whorl of the rosette for all leaf measurements. We measured two perpendicular diameters of the plant, then averaged them to obtain a single value. A 15 cm-long section was cut from the middle part of the leaf, preserved in bags with wet paper, and taken to the laboratory to take four measurements associated with the lateral teeth. Leaf color was recorded using the Munsell color system for plant tissues [39]. In addition, we calculated eight relationships between the raw variables, resulting in a total of 25 variables. We carried out multivariate analyses to examine the morphological characteristics of the agave varieties according to the proposed management categories. Statistical analyses were carried out using the R programming language [40]. Due to the different types of characters and units of measurement, we standardized the data matrix using the scale function (mean-centered). We then carried out a Principal Component Analyses (PCA) and constructed a PCA dendrogram and heatmap using Ward’s minimum variance method. Discriminant Function Analysis (DFA) was performed using JMP software [41].

### Sample collection and DNA extraction, amplification, marker screening and data quality

In total, 127 samples of agave tissue were collected from healthy leaves (details in Tables 2 and 5), dried, and stored with silica gel until DNA extraction [42]. DNA was extracted using the CTAB method [43], purified with chloroform: octanol (24:1) and resuspended in TE buffer. To quantify DNA concentration, 260 and 280 nm absorbance readings were done on a NanoGenious spectrophotometer (MAPADA Instruments Co., Ltd., 2017). Sixteen nuclear microsatellite loci designed for *Agave* species were evaluated (Table 1) [44,45]. PCR reactions were performed using an ARKTIK thermal cycler (Thermo Scientific), under the following conditions: initial denaturation at 95°C for 5 min, 30 denaturation cycles at 95°C for 45 s, alignment according to Table 1 for 45 s, and extension at 72°C for 40 s, followed by a final incubation at 72°C for 5 min. The volume of the reaction mixture was ~ 8 μL containing 10–50 ng (1.5 μL) of genomic DNA, 25 mM (1.6 μL) of MgCl_2_, 1–10 μM (0.6–1 μL) of forward and reverse primers, 10 mM (0.3 μL) of dNTP mix, 2.3 μL of buffer (5x), 0.5 μL of DNAse-free water and 1.5U (0.3 μL) GoTaq® Flexi DNA Polymerase (Promega). The PCR products were resolved on a 15% polyacrylamide gel stained with ethidium bromide (10 mg/mL) run for 90 min at 90 V.

**Table 1:** Nuclear microsatellites designed for agave species that were amplified in this study.

Locus	Allele size range reported (bp)	Allele size range in this study (bp)	Annealing temperature (°C)	Reference
APAR2-12	151–205	147–167	63.8	Lindsay et al. 2012 [44]
APAR3-11	158–194	155–163	63.4	
APARLC20	204–240	203–239	59.2	
APARLC21	142–206	142–178	56.6	
APARLC28	138–195	183–197	60	
APARLC34	152–206	160–175	64	
APARLC35	157–175	158–188	56	
BYU3268	138–147	136–145	57.2	Byers et al. 2014 [45]
BYU3674	147–156	144–156	59.1	
BYU4012	131–140	132–138	60	
BYU4463	178–199	172–190	60	
BYU4988	172–190	167–205	57	
BYU5164	155–176	153–181	64	
BYU7269	155–167	155–171	64	
BYU8490	173–191	163–184	60	
BYU8677	171–198	170–192	59	

The size ranges, reported base pairs, are provided both from previous reports (see Reference column) and those recovered in this study. The annealing temperature for each locus is shown.

Alleles (bands) were scored using GelAnalyzer 2010a [46]. We choose the most intense and defined bands to be interpreted as codominant diploid data. After genotyping, the data set was analyzed in Micro-Checker 2.2.3 [47] to determine the presence of null alleles for each locus with 1000 bootstrap simulations and a confidence interval (CI) with Bonferroni’s correction and Chakraborty’s estimator [48]. Deviations from Hardy-Weinberg equilibrium (HWE) was tested using the package “Pegas” in R [40,49]. To check for non-random association among loci, we tested for linkage disequilibrium (LD) in Arlequin ver. 3.5 [50].

### Genetic diversity

The descriptive diversity statistics—the percentage of polymorphic loci (PPL), mean allele diversity (A), effective alleles (Ae), observed and expected heterozygosity (H_O_ and He), and genetic diversity—were calculated using the R package “Poppr” [40,51] at the level of traditional varieties and management categories.

#### Genetic structure

The global and pairwise F_ST_ at the level of traditional varieties and management categories were determined using the FreeNA program, which corrects for bias due to the presence of null alleles using the ENA method with 10,000 bootstrap repetitions [52]. The inbreeding coefficient (F_IS_) was calculated, correcting for null alleles, with the INEst program [53] using the Bayesian model IIM assuming inbreeding. Each run consisted of 10,000 burn-in and 50,000 periods of Markov Chain Monte Carlo simulations (MCMC). A neighbor-joining tree was constructed based on Nei’s genetic distance [54] using 10^3^ bootstrap replications in Poptree2 software [55] at the level of traditional varieties. To analyze the genetic structure, we used STRUCTURE v 5.4 under the admixture and correlated allele frequency models [56]. Ten independent runs of 50,000 Markov chain Monte Carlo (MCMC) replications with a burn-in of 50,000 runs for each K-value varying from 1 to 15 groups (*K*), were performed [57]. The appropriate allocation limit to the number of groups with the lowest accumulated variance was calculated using the graphic method proposed by Evanno et al., [58] generated from the StructureSelector on the web (https://lmme.qdio.ac.cn/StructureSelector/ [59] at the level of traditional varieties. Multivariate statistical approaches using discriminant analysis of principal components (DAPC) were performed and plotted in R using the package “adegenet” at the level of traditional varieties [40,60]. For the multivariate analyses, only the traditional varieties from the *Salmianae* group were included, and we excluded varieties that were represented by a single individual.

## Results

### Pulque agave production systems and management categories

In *El Cubo*, the producers have small plots that have been used for producers for at least two generations. The agaves are cultivated in *hileras* (rows) interspersed with other crops or as living fences within producers’ properties. The CCUB1 plot is a production system measuring 3.529 ha, where agaves are cultivated in rows within fields of seasonal crops, such as maize and beans. The CCUB2.1 plot is a backyard orchard with fruit trees and ornamental plants, occupying an area of 0.35 ha. In the CCUB2.2 and CCUB2.3 plots, agaves are cultivated in rows among crops, such as maize and beans, and occupy a space of 1.309 and 0.781 ha, respectively, belonging to a single producer of Hñähñu origin. The agaves are propagated by transplanting 50- to 100 cm-tall suckers from the agaves from the plot into rows. Agaves purchased from other producers in the area are also occasionally incorporated into the plots.

**Fig 2:**
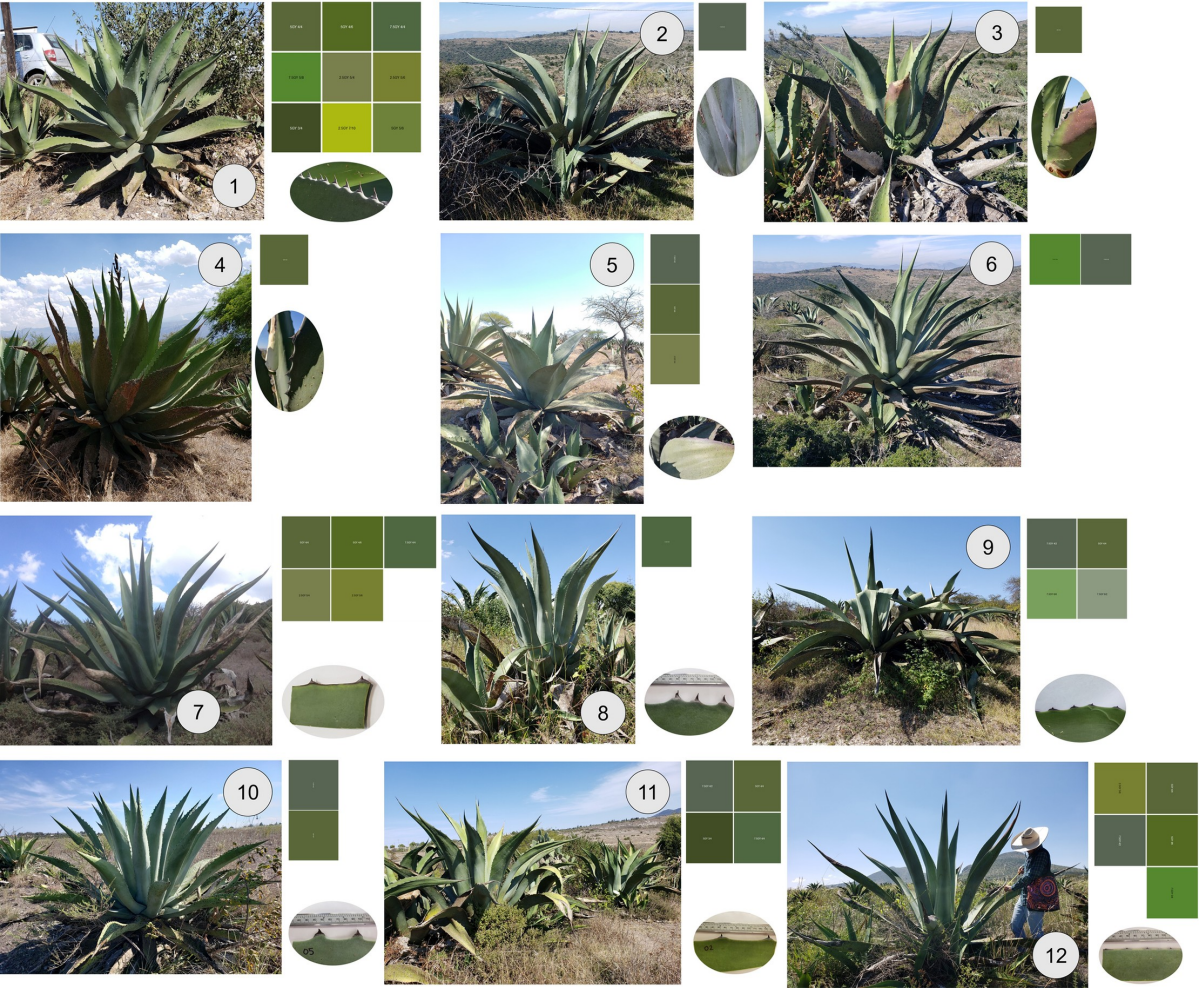
Traditional varieties of pulque agave recorded in the study localities in the state of Hidalgo 1) *Corriente*, 2) *Corriente cenizo*, 3) *Corriente colorado* 4) *Corriente espina china*, 5) *Corriente penca ancha*, 6) *Corriente penca larga*, 7) *Manso de zoqui*, 8) *Xaminí*, 9) *Poblano*, 10) *Guanté*, 11) *Mutá*, 12) *Penca Larga*.

Agave suckers are classified for sale according to their vigor into first-rate plants, sold for about 50 Mexican pesos (2 USD) each, and second- and third-rate plants that sell for about 30 Mexican pesos (1.2 USD). Some families in the area sell their plants to pulque producers and *barbacoyeros* (people who use agave leaves to cook a regional dish: *barbacoa de borrego*) for 100 Mexican pesos (4 USD) per plant. In this locality we recorded eight traditional varieties of agave used for pulque production. There was one wild-growing variety, known as 1) *Corriente*, or *Bronco* or *Verde de monte*, and seven cultivated varieties: 2) *Chino*, 3) *Xaminí*, 4) *Penca larga*, 5) *Mutá*, 6) *Hoc´uadá*, 7) *Poblano*, and 8) *Guanté* (also known as *Maguey blanco*) (Table 2, Fig 2). The producers affirm that their ancestors frequently used and collected sap from wild-growing agaves (*Corriente*), but that they currently use cultivated agaves.

**Table 2:** Characteristics of traditional varieties of agaves identified by producers in the two study localities in Hidalgo state.

Traditional variety name (n = individuals measured)	Main Ethnobotany Features	Managed category	Location	Taxonomic identity	Voucher specimen
*1*. *Corriente*, or *bronco*, or *verde de monte* (Morphological analysis, n = 41. Genetic analysis, n = 63)	This variety is a wild species that is also managed in some localities. A medium to small plant with large and abundant lateral teeth. Produces abundant suckers. The roots are strong, so some prefer to use it for living fences, it is extremely easy to transplant. Currently it is generally not used for *aguamiel* because it produces a low volume per day, produces for fewer days, and sap is of lower quality (less sweet). It is also used for *xanté* (agave fiber), although this use is decreasing. It produces a high degree of *guixe* (contact dermatitis from sap).	WCO, T, TR	Morphological analysis: CCOY1 (n = 9), WCOY2 (n = 14), WSAUZ(n = 18) Genetic analysis: CCOY1 (n = 11), WCOY2 (n = 34), WSAUZ (n = 18)	*Agave salmiana* ssp. *crassispina*	CJFU010 CJFU011 CJFU015 CJFU016
*2*. *Corriente cenizo (n = 2)*	Similar in characteristics to the *Corriente* variety, but leaf color is glaucous.	WCO	WCOY2	*Agave salmiana* ssp. *crassispina*	Photo record
*3*. *Corriente colorado (n = 1)* *Not included in genetic analysis*	Similar in characteristics to the *Corriente* variety, but it is smaller, with reddish leaves. Produces very little *aguamiel*.	TR	CCOY1	*Agave salmiana* ssp. *crassispina*	Photo record
*4*. *Corriente espina china (n = 1)* *Not included in genetic analysis*	This variety is similar to the *Corriente* variety but has more pronounced leaf margins greater size and abundance of lateral teeth.	TR	CCOY1	*Agave salmiana* var. *ferox*	Photo record
*5*. *Corriente penca ancha (n = 6)*	Like *Corriente*, but was wider, more flexible, and more fibrous leaves. The leaves of this variety are used to prepare *barbacoa*.	WCO	WCOY2 (n = 4), WSAUZ (n = 2)	*Agave salmiana* ssp. *crassispina*	Photo record
*6*. *Corriente penca larga (n = 7)*	Remarkably similar to *Corriente* but has a longer *penca* (leaf) and produces sweet *aguamiel*.	T, TR	CCOY1	*Agave salmiana*	CJFU012 CJFU013
*7*. *Manso de zoqui* or *maguey fino (n = 21)*	This variety is purchased in Zoquizoquipan, Metztitlán municipality. It has been grown for more than 40 years in the region. It is the largest variety and is highly valued because it produces a large amount of sweeter *aguamiel*. It can produce up to four liters a day for up to five months.	C	CCOY1	*Agave salmiana* var. *salmiana*	CJFU014
*8*. *Xaminí* or *Xa`mini (n = 1)* *Not included in genetic analysis*	This is a native variety from the *Valle del Mezquital*. It is of medium size is characterized by hook-shaped lateral teeth (to which the name *Xaminí* in Hñähñu refers; can also translated as “spike that scrapes”). More lateral teeth. Grows fast and is ready for harvest in 9 to 10 years. The sweetest and least viscous *aguamiel* called *aguamiel clarito*.	C	CCUB2.3	*Agave salmiana* ssp. *crassispina*	CJFU007
*9*. *Poblano* (n = 12*)*	Arrived in the region 22 years ago through a state government program. The plants are long and have wide leaves (*pencas)*. It is very susceptible to pests such as *pinacatillo* (maguey weevil). Produces a large amount of *aguamiel*.	C	CCUB1 (n = 10), CCUB2.3 (n = 2)	*Agave salmiana* var. *salmiana*	CJFU005
*10*. *Guanté* or *maguey blanco (n = 3)* *Not included in genetic analysis*	Cultivated species. Has some morphological similarities with *Xaminí*. It takes 15 years for the stalk to emerge, which is about two meters high.	C	CCUB2.1 (n = 1), CCUB2.2 (n = 2)	*Agave aff*. *americana*	CJFU008
*11*. *Mutha*, or *mutá (n = 9)*	Has a wide *penca* (leaf), it is larger than the *Corrientes*. Their *pencas* (leafs) are used for *barbacoa*. Takes about 12 to 13 years to be ready to harvest.	C	CCUB2.1 (n = 4), CCUB2.2(n = 3), CCUB2.3 (n = 2)	*Agave salmiana* var. *salmiana*	CJFU006
*12*. *Penca larga (n = 4)*	It is a large variety, with long leaves and slightly rigid, takes about 14 years to be ready for harvest. *Aguamiel* is more viscous than other varieties. It used to take *ayate* (fiber). This is not good for *barbacoa*. Does produce *aguamiel*, but it is more viscous, so *pulque* from this variety is thicker and spoils quickly.	C	CCUB2.2 (n = 1), CCUB2.3 (n = 3)	*Agave mapisaga*	CJFU002

Wild collected (WCO), Tolerated (T), Transplanted (TR), Cultivated (C).

In this locality, each producer uses six to 20 plants simultaneously. The sap is collected with the elongated tool known as an *acocote*, essentially a large straw. One end is inserted into the cavity of the agave, and suction is generated by mouth at the other end, drawing the sap into the *acocote* which is then emptied into a bucket. *Acocotes* were traditionally made with a long, dried Cucurbitaceae fruit (*Lagenaria siceraria*), however due to the low availability of this material and its fragility, it has been replaced by plastic materials. Currently, *acocotes* are made by attaching a plastic hose (the end inserted into the plant) to a plastic bottle, which is drilled at the base (to allow suction). Pulque is produced with a combination of different sap varieties. The producers mix the fresh sap with old pulque, produced the day before. The fresh sap and pulque are used for self-consumption and sold locally at 10 Mexican pesos (0.4 USD) per liter, or to tourists for 25 Mexican Pesos (1 USD) per liter.

*La Coyotera* has been cultivating agaves for around 30 years. A total of 44 hectares are dedicated to agave production, where the plants are found at different densities and are managed in three ways: living fences, *jilas*, and nursery. For living fences, used to delimit spaces and paths, the *Corriente* variety is preferred, since it has more teeth (Total number of teeth-TEET), longer spines (Terminal thorn length), is fast growing, and has deep roots. *Jilas* refers to rows of agaves that are interspersed with native vegetation and are planted perpendicular to the slope of the land. Juvenile agaves are cultivated and kept in nurseries to protect them from goat browsing and allow them to develop and are then transplanted into the *jilas* when they reach 100 cm in height.

The cultivated agaves come from various sources, including from the suckers of agaves from nearby areas and suckers bought from local producers and from other productive areas such as Zoquizoquiapan, Zotoltepec and Singuilucan in Hidalgo and Magdalena Contreras in Mexico City. In this locality, agaves are also used to produce pulque. Fourteen traditional varieties used for pulque production were listed: 1) *Corriente*, 2) *Corriente cenizo*, 3) *Corriente colorado*, 4) *Corriente espina china* 5) *Corriente penca ancha*, 6) *Corriente penca larga*, 7) *Manso de zoqui*, 8) *Manso del altiplano*, 9) *Xaminí*, 10) *Púa larga*, 11) *Verde*, 12) *Penca larga* 13) *Blanco* and 14) *Sabililla* (Table 2, Fig 2). In this locality, 35 plants are used simultaneously; collecting, and preparing pulque similarly to the other locality and the price per liter is 10 Mexican pesos (0.4 USD).

In both localities there is a gradient of management practices that can be grouped into the following categories:

1. Wild collected (WCO). Agaves that inhabit the surrounding areas of native vegetation. These plants are collected *in situ* and used for various purposes. According to the people interviewed, some 70 years ago there were no crops, and these agaves were used by wild collection.
2. Tolerated (T). Agaves that were already growing wild in an area of native vegetation that was later converted to a productive system and have been maintained by the managers within the new system. These plants receive some management, such as removing dry leaves.
3. Transplanted (TR). Agaves removed from their original place, generally from nearby ecosystems, and placed within productive systems, forming part of the rows and living fences.
4. Cultivated (C): Agaves cultivated within productive systems. This category generally contains the most used and valued traditional varieties. These are given greater care, including pruning and more intense propagation. These are the varieties that are bought or exchanged between localities.

Producers identified qualitative characteristics that make it possible to distinguish *cerro* and *monte* agaves (wild-collected) from cultivated ones. These characteristics are the size of the plant and its leaves, the shape and length of the spines, and the number of lateral teeth. Another important characteristic for managers is the color of the plant, which ranges from different shades of green to glaucous. Characteristics such as the time to reproductive maturity, the sap quantity, and qualities (such as sweetness or viscosity), and the duration of sap production are also considered (Table 2). Wild-collected agaves can be distinguished from cultivated agaves because they are not aligned, have a smaller rosette with many lateral teeth, and are larger than cultivated ones. Of the traditional varieties described in Table 2, three were found in natural ecosystems and were wild-collected (WCO), two were tolerated within production systems (T), four varieties were transplanted from native vegetation to managed areas (TR) and six varieties are cultivated (C). The traditional variety *Corriente* was the most abundant and can be found in most of the management categories.

### Morphological variability of pulque agaves

**Fig 3:**
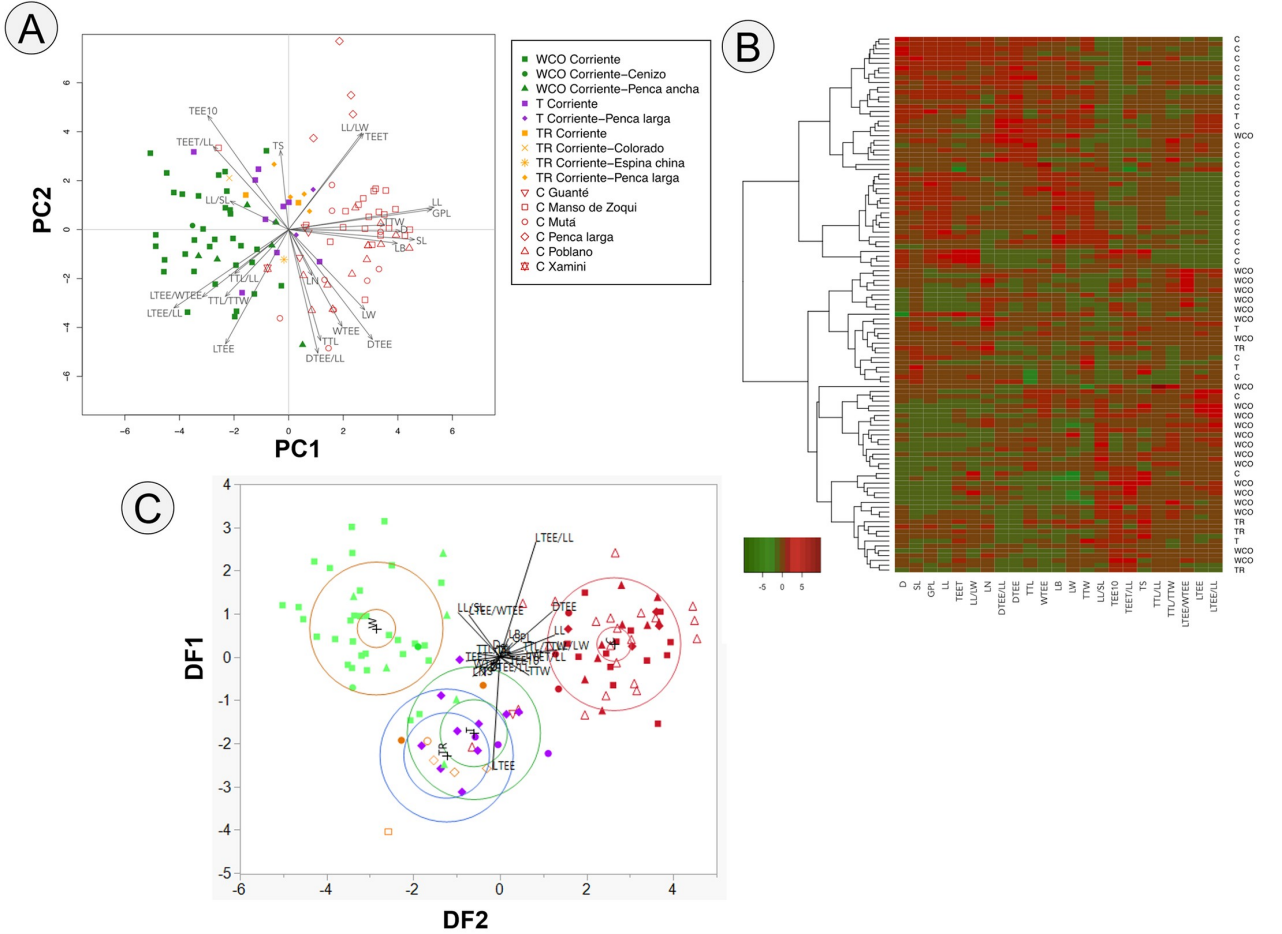
A) Principal components analysis (PCA) of the 23 morphological variables measured in the twelve traditional varieties of *Agave* in the studied localities in Hidalgo state. The wild-collected agaves are in green, tolerated in purple, transplanted in yellow and cultivated in red. B) Heatmap of management categories of agave and morphological traits (Ward’s minimum variance method). Two large groups are presents, the first containing the cultivated agaves, and the second containing the wild-collected agaves and the majority of tolerated and transplanted agaves. Characteristics related to the gigantism of the individual are associated with cultivated varieties and increased thorniness was associated with wild-collected agaves. C) Discriminant analysis for all management categories, wild collected (symbols in green colors), Transplanted (symbols in orange colors), Tolerated (symbols in purple colors) and, Cultivated (symbols in red colors) DF1 83.46% and DF2 12.58%.

Of the 19 traditional varieties recorded, it was only possible to morphologically characterize 12 varieties because no measurable individuals were found of the other seven. Based on 23 morphometric variables, the first two PCA components together explained 47% of the variance (PC1: 29%, PC2: 18%; Fig 3A). The ordering of PC1 was related to the size of the plant, and the most important variables were leaf length (LL), General plant length (GPL), stem length (SL), the ratio of the length of lateral teeth to the length of the blade (LTEE/LL), and diameter (D). In PC2, the most important variables were associated with the plant’s thorniness: the ratio of the distance between teeth and the length of the leaf (DTEE/LL), the length of the lateral teeth (LTEE), number of teeth per 10 cm (TEE10), the length of the terminal spine (TTL), and the distance between teeth (DTEE) (Table 3). The agaves of the different varieties sorted by management category, with wild-collected agaves found on the extreme left of the arrangement and cultivated in the right, while the transplanted and tolerated individuals were located in the center (Fig 3A).

**Table 3:** Vegetative morphological characteristics measured in the traditional varieties of agaves recorded in the localities *Rancho La Coyotera* and *El Cubo* studied in the state of Hidalgo.

Vegetative character	PC1	PC2	DF1	DF2
General plant lenght (GPL)	**0.350**	0.055	0.302	0.339
Stem lenght (SL)	**0.308**	-0.027	0.016	0.096
Mean diameter of the plant (D)	**0.274**	-0.005	-0.135	0.165
Leaf lenght (LL)	**0.355**	0.061	**1.492**	**0.598**
Leaf number (LN)	0.057	-0.125	-0.425	-0.303
Thickness leaf (TS)	-0.020	0.216	-0.290	-0.295
Suckers (S)	not included in the analysis	not included in the analysis	-0.127	-0.220
Leaf width at middle (LW)	0.186	-0.219	0.416	-0.039
Leaf width at base (LB)	0.266	-0.037	0.160	0.338
LL/LW	0.179	0.264	**1.054**	0.131
LL/SL	-0.143	0.078	**-0.721**	**0.768**
Terminal thorn length (TTL)	0.078	**-0.303**	-0.054	0.038
Terminal thorn width at the base (TTW)	0.235	0.013	**0.554**	-0.349
Terminal thorn distance a first tooth (TTL-TEE)	not included in the analysis	not included in the analysis	not included in the analysis	not included in the analysis
TTL/TTW	-0.155	-0.182	0.406	0.109
TTL/LL	-0.132	-0.120	-0.368	0.053
Total number of teeth (TEET)	0.183	0.262	**-0.583**	-0.070
TEET/LL	-0.184	0.227	0.454	-0.076
Number of teeth in 10 cm^2^ (TEE10)	-0.198	**0.311**	0.167	-0.138
Teeth length (LTEE)	-0.154	**-0.312**	-0.118	**-1.805**
LTEE/LL	**-0.280**	-0.214	**0.678**	2.174
Teeth width (WTEE)	0.131	-0.264	-0.402	-0.186
LTEE/WTEE	-0.211	-0.184	**-0.560**	**0.761**
Distance between teeth (DTEE)	0.205	**-0.299**	**0.956**	**0.835**
DTEE/LL	0.072	**-0.337**	-0.150	-0.241
Color (C)	not included in the analysis	not included in the analysis	not included in the analysis	not included in the analysis

The columns show eigenvectors of the first (PC1) and second (PC2) principal components according to PCA and eigenvectors of the first (DF1) and second (DF2) factors of the DFA. Bold type indicates the characteristic with the strongest contribution to ordination.

The heatmap showed that the agaves were grouped according to the management category. Two large groups were observed, the first one in the upper part composed mainly of cultivated agaves, and the second group mostly containing wild-collected agaves. The attributes that distinguished them were related to domestication syndrome (gigantism and thorniness). The cultivated agaves (top in the heatmap) had larger size and were less thorny, regardless of their variety. They had smaller lateral teeth and the teeth were smaller proportionally to the leaf. This first group of cultivated agaves was divided into two subgroups: the first one made up of C and some WCO and T individuals that, although they belong to other management categories, present some morphological characters with tendencies towards domestication syndrome and represent those agaves that are progressively selected for possessing these desirable characteristics, such as larger size (*Corriente penca ancha*). The other subgroup was composed exclusively of cultivated agaves, highlighting the presence of individuals of the variety *Penca larga* (*A*. *mapisaga*), which represent the most marked domestication syndrome. It should be noted that it is a species whose wild ancestor is unknown, and it is a species that is exclusively under cultivation.

The second large group was composed mainly of WCO agaves. This group was also divided into two subgroups. There was a greater abundance of the WCO agaves, as well as some of the T and TR. These groups also contained some C agaves with characteristics that are not so desirable for producers, such as greater thorniness and smaller size compared to the agaves located at the top of the heatmap, such as the cultivated variety *Guanté* (*A*. *americana*), which represents a species It distinguishes the Salmianae group and there it is located with greater morphological similarity with the WCO agaves, since it is not a species of size as large as *Agave salmiana* var. *salmiana* or *A*. *mapisaga*. The group at the bottom of the heatmap contained the agaves with the highest values of variables related to thorniness, such as high number of teeth in relation to the size of the leaf (Fig 3B).

Regarding the characteristics associated with gigantism, the variety *Corriente* (*A*. *salmiana* ssp. *crassispina*) was the smallest in terms of GPL, SL, D, and LL. The cultivated variety *Penca larga* (*A*. *mapisaga*) was the largest in terms of GPL, D and LL, while the cultivated variety *Poblano* (*A*. *salmiana* var. *salmiana*) had the highest average SL. In terms of thorniness, *Penca larga* had more teeth and more closely spaced teeth than the other varieties, but the size of the teeth was smaller than the wild varieties. The variety *Manso de zoqui* also had a smaller tooth size. This confirms the domestication syndrome of lesser dentition for easier manipulation (Table 4). Carrying out the morphological comparisons among the management categories, we found that the wild-collected category was the smallest (GPL, SL, D, LL) and cultivated the largest. Wild-collected agaves had larger lateral tooth sizes, but not a larger Terminal thorn (TTL), which was similar among all management categories (Table 5).

**Table 4:** Vegetative morphological characteristics of eight traditional varieties of pulque agave, identified by producers in the localities of *La Coyotera* and *El Cubo*, in the state of Hidalgo.

Vegetative character	Traditional varieties of agave							
	*Corriente*	*Corriente penca ancha*	*Corriente penca larga*	*Manso de zoqui*	*Poblano*	*Guanté*	*Mutá*	*Penca larga*
General plant length (GPL)	172.955±4.185	194.000±7.625	218.714±6.578	256.286±8.050	252.308±8.209	225.333±6.227	240.333±10.813	260.000±10.206
Stem length (SL)	42.898±2.520	50.000±5.882	80.243±5.096	68.829±3.233	81.692±4.532	71.667±3.333g	78.444±3.271	66.750±3.728
Mean diameter of the plant (D)	253.523±6.498	271.417±13.679	303.000±10.341	321.024±9.249	368.692±14.644	331.500±4.770	296.944±45.123	369.500±8.663
Leaf length (LL)	116.561±2.801	132.167±5.089	143.857±4.748	188.438±6.138	185.154±6.052	148.000±2.000	180.000±5.336	209.750±11.213
Terminal thorn length (TTL)	5.051±0.165	5.554±0.319	4.138±0.611	5.408±0.224	6.069±0.339	3.713±1.408	6.479±0.462	3.225±0.183
Number of teeth in 10 cm^2^ (TEE10)	3.636±0.187	2.667±0.333	3.143±0.340	2.429±0.177	2.000±0.000	2.000±0.000	2.000±0.167	4.750±0.629
Teeth length (LTEE)	0.769±0.043	0.750±0.042	0.716±0.057	0.469±0.027	0.794±0.060	0.699±0.121	0.768±0.066	0.301±0.029
LTEE/LL	0.007±0.000	0.006±0.000	0.005±0.000	0.003±0.000	0.004±0.000	0.005±0.001	0.004±0.000	0.001±0.000
Distance between teeth (DTEE)	1.687±0.015	2.595±0.597	2.020±0.304	3.058±0.254	4.509±0.277	4.389±0.618	4.892±0.698	0.778±0.066
DTEE/LL	0.015±0.0	0.020±0.0	0.014±0.0	0.017±0.0	0.025±0.002	0.030±0.004	0.028±0.004	0.004±0.001
Allele richness (A)	6.000±0.428	3.313±0.285	2.813 ± 0.306	3.500 ± 0.316	3.313 ± 0.435	1.563 ± 0.182	3.063 ± 0.452	2.313 ± 0.237
Allele effective (Ae)	4.073 ± 0.303	2.590 ± 0.221	2.226 ± 0.238	2.390 ± 0.235	2.495 ± 0.345	1.459 ± 0.146	2.294 ± 0.347	1.975 ± 0.193
Observed Heterocigozity (Ho)	0.190 ± 0.052	0.167 ± 0.050	0.161 ± 0.070	0.143 ± 0.056	0.214 ± 0.086	0.146 ± 0.074	0.203 ± 0.083	0.125 ± 0.065
Expected Heterocigozity (He)	0.730 ± 0.023	0.562 ± 0.046	0.493 ± 0.040	0.517 ± 0.048	0.487 ± 0.059	0.219 ± 0.065	0.435 ± 0.066	0.410 ± 0.061
Inbreeding coefficient (F_IS_) (CI)	0.716 (0.680–0.719)	0.702 (0.565–0.709)	0.562 (0.378–0.575)	0.643 (0.523–0.647)	0.159 (0.150–0.275)	0.194 (0.003–0.228)	0.324 (0.077–0.326)	0.650 (0.353–0.656)

**Table 5:** Vegetative morphological characteristics according to the management categories of the eight traditional varieties of pulque agaves, identified by producers in the localities of *La Coyotera* and *El Cubo*, in the state of Hidalgo.

Vegetative character	Management category			
	Wild collected (WCO) (n = 60)	Tolerated (T) (n = 13)	Transplanted (TR) (n = 8)	Cultivated (C) (n = 49)
General plant length (GPL)	170.850±5.085	202.455±9.646	207.875±11.311	246.615±4.436
Stem length (SL)	40.025±2.459	64.909±4.689	68.963±5.498	73.460±2.156
Mean diameter of the plant (D)	249.900±9.275	289.091±17.687	294.938±20.720	328.603±8.135
Leaf length (LL)	115.200±3.792	135.273±7.232	142.500±8.480	181.998±3.326
Terminal thorn length (TTL)	5.049±0.210	4.777±0.400	4.473±0.469	5.479±0.184
Number of teeth in 10 cm^2^ (TEE10)	3.450±0.178	3.091±0.339	3.750±0.398	2.500±0.156
Teeth length (LTEE)	0.800±0.041	0.713±0.077	0.660±0.091	0.622±0.036
LTEE/LL	0.007±0.000	0.005±0.001	0.005±0.00	0.004±0.000
Distance between teeth (DTEE)	1.851±0.216	2.099±0.411	1.567±0.482	3.556±0.189
DTEE/LL	0.016±0.001	0.015±0.003	0.012±0.003	0.020±0.001
Allele richness (A)	5.587 ± 0.407	2.250 ± 0.348	3.063 ± 0.347	5.188 ± 0.572
Allele effective (Ae)	3.562 ± 0.221	2.386 ± 0.281	2.389 ± 0.255	3.418 ± 0.572
Observed Heterocigozity (H_O_)	0.187 ± 0.048	0.204 ± 0.079	0.135 ± 0.064	0.167 ± 0.058
Expected Heterocigozity (He)	0.701 ± 0.020	0.495 ± 0.055	0.504 ± 0.053	0.646 ± 0.040

The DFA explained 96.03% of the variance (DF1 83.46%; DF2 12.58%). Three large groups were differentiated; one contained the wild collected varieties (left side of Fig 3C), a second was composed of cultivated traditional varieties (right side of Fig 3C), and a third group contained the tolerated and transplanted traditional varieties (center bottom). The variables with the highest eigenvalues in DF1 were LL, LL/LW, LL/SL, TTW, TEET, LTEE/LL, LTEE/WTEE and DTEE. In the case of DF2, they were LL, LL/SL, LTEE, LTEE/WLEE, and DTEE. These characteristics are associated with the length of the leaves and the dentition. The first group, in the upper left it was made up of wild-collected individuals of the traditional varieties *Corriente*, *Corriente cenizo* and *Corrriente penca ancha*. The second group (top right) was made up of individuals cultivated from traditional varieties *Penca larga*, *Manso de zoqui*, *Mutá* and *Poblano*. The third group (bottom center) corresponded to tolerated and transplanted individuals of different varieties (their centroids did not differ), even wild and cultivated individuals are distinguished. Wilk’s Lambda had a value close to zero (0.05; *p* < 0.001), indicating that the information provided by the variable was statistically significant, allowing the discrimination of groups whose centroids are not same (wild collected and cultivated) and have little overlap. Only 7% of the individuals were not correctly classified into the category management assigned *a priori*. 90% of the individuals in the management category WCO were classified correctly; 7.5% were classified T and 2.5% to TR. 85% of the individuals in the T category were classified correctly and 15% classified as TR. All individuals in the TR category were correctly classified. In the case of management category C, 96% were classified correctly and 4% to category T.

### Genetic diversity and structure

**Fig 4:**
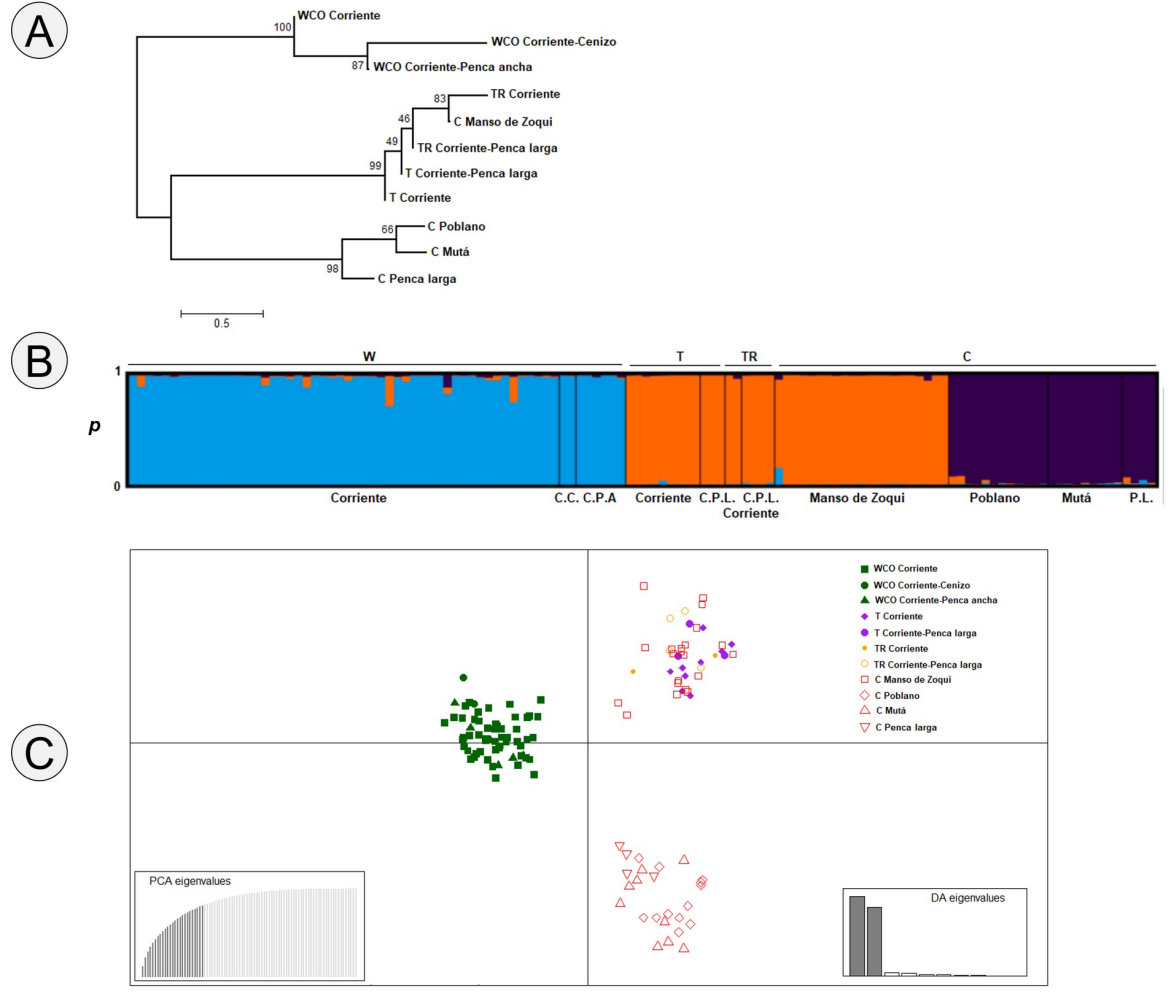
A) Neighbor-joining tree of traditional varieties of *Agave* in Hidalgo state constructed using Nei’s genetic distances. The numbers shown are bootstrap probabilities based on 10,000 replicates. The length of the bar is equal to a genetic distance of 0.05. B) Bayesian model-based clustering STRUCTURE analysis as inferred with K = 3 (C.C: *Corriente cenizo*; C.P.A.: *Corriente penca ancha*; C.P.L.: *Corriente penca larga;* P.L: *Penca larga*, C) Multivariate approach DAPC, the eigenvalues from the Discriminant Analysis are shown.

Null alleles were found at 16 loci, suggesting homozygote excess. Only one locus (BYU4463) did not exhibit null alleles. The estimated null allele frequencies over traditional varieties and managed category varied from -0.0738 (7%) at BYU4463 (this suggest heterozygote excess) to 1 (monomorphic loci) at APAR3-11, APARLC28, APARLC34 and BYU 4012. Sixteen loci showed departures from HWE (*p* < 0.05). LD was observed between four pairs of loci (*p* <0.05: APAR2-12 x APAR3-11, APARLC-21 x APARLC-28, APARLC-34 x BYU3674 and APARLC-35 x BYU4012. The percentage of polymorphic loci (PPL) in traditional varieties ranged from 43.75% (*Guanté*) to 100% (*Corriente*, *Corriente penca larga* and *Manso de zoqui*). In the management categories, this was 100% for wild collected (WCO) and cultivated (C) and 93.75% for tolerated (T) and transplanted (TR). The allele richness (A) in the traditional varieties ranged from 1.563 (*Guanté*) to 6 (*Corriente*), and the effective number of alleles (Ae) varied from 1.459 to 4.076 in the same traditional varieties (*Guanté* and *Corriente* respectively; Table 4). In the managed categories, the allele richness ranged from 3.063 (TR) to 5.875 (WCO), and the effective number of alleles (Ae) was 2.386 to 3.562 in the same managed categories (TR, T and WCO, Table 5). The observed heterozygosity (Ho) in traditional varieties ranged from 0.125 (*Penca larga*) to 0.214 (*Poblano*) and the expected heterozygosity (He) varied from 0.219 (*Guanté*) to 0.730 (*Corriente*) (Table 4). By management category, the observed heterozygosity (H_O_) ranged from 0.135 (TR) to 0.204 (T) and the expected heterozygosity (He) varied from 0.495 (T) to 0.701 (W, Table 5). Average levels of genetic variation at the species level for *Agave salmiana* was (Hs = 0.564, N = 7), for *A*. *salmiana* ssp. *crassispina* (Hs = 0.738; N = 73), for *Agave salmiana* var. *salmiana* (Hs = 0.540, N = 21); for *A*. *mapisaga* (Hs = 0.526, N = 4) and for *A*. *americana* (H_S_ = 0.291, N = 3). F_ST_ with ENA correction was 0.127, indicating a moderate genetic differentiation among management categories. F_IS_ ranged from 0.194 (*Guanté*) to 0.720 (*Corriente*), indicating strong inbreeding. The neighbor joining tree based on Nei’s distances showed three groups. The first included the traditional varieties: WCO *Corriente*, WCO *Corriente cenizo* and WCO *Corriente penca ancha*. The second group contained TR *Corriente*, C *Manso de zoqui*, TR *Corriente penca larga*, T *Corriente Penca larga* and T *Corriente*, and the third contained the C *Poblano*, C *Mutá*, C *Penca larga* varieties (Fig 4A). The Bayesian cluster analysis indicated that the most likely number of genetic groups was three (K = 3, Fig 4B). The blue genetic group corresponds to the category of wild-collected and the traditional varieties *Corriente*, *Corriente cenizo* and *Corriente penca ancha*. The orange group is made up of individuals that are tolerated, transplanted, and cultivated, with the traditional varieties *Corriente*, *Corriente penca larga*, and *Manso de zoqui*. The purple group corresponds to the category of cultivated management and the traditional varieties *Poblano*, *Mutá* and *Penca larga*. This analysis was consistent with the grouping of the dendrogram. The DAPC also grouped the plants according to management categories, left in the middle the wild collected traditional varieties (Fig 4C) matched the dendrogram and Bayesian clustering. In the lower right, a mixed group containing tolerated and transplanted traditional varieties together with a cultivated traditional variety *Manso de zoqui*. In the lower left, there was a group formed by the cultivated traditional varieties (*Poblano*, *Guanté*, *Mutá*, *Penca larga*).

## Discussion

A rich diversity of traditional varieties of pulque agave are used in the localities studied in the state of Hidalgo, which are maintained in a management gradient in traditional agroforestry systems. The cultivated plants clearly exhibited domestication syndrome related to gigantism and reduced thorniness. We also found support for the hypothesis of decreased genetic diversity and moderate population structure among cultivated pulque agaves compared to wild populations.

### Agrobiodiversity of pulque agave in the State of Hidalgo

In Mexico, about 117 agave taxa have been reported to be used for various purposes, which have been assigned about 570 common names and represent varieties of agronomic interest [61]. We found a high diversity of traditional varieties of pulque agave, which are compared with those recorded by other authors in other regions of the country. Although there is a wide agrobiodiversity of pulque agaves, they belong to only three species: *Agave americana*, *A*. *salmiana* and *A*. *mapisaga*. The assignment of taxonomic identities in agaves is a complex task. Some of the species descriptions are based on *ex situ* specimens, and agaves are a group of plants where hybridization is common, even more so in cultivated species and varieties. In our case, the plants managed in crops are difficult to identify at the species level because reproductive structures are frequently removed as part of management, so these structures are not available to aid in identification.

In the southern highlands, Mora-López et al. [23] recorded 62 varieties of pulque agaves. In the state of Hidalgo, Reyes-Agüero et al. [36] found that the mestizo and Hñahñü communities of the Mezquital Valley manage around 15 and 21 varieties, respectively. In our case, we recorded similar numbers of varieties with the same trends as Reyes-Agüero et al. [36], since the production system managed by the oldest producer of Hñahñü origin maintains unusual traditional varieties that were not recorded in other production systems. In the state of Puebla, Álvarez-Duarte et al. [25] recorded seven varieties of pulque agaves and, unlike previous studies, producers in this area maintain the species *A*. *applanata* among their crops, which was widely used in the past. In the state of Tlaxcala, nine varieties of pulque agaves have recently been identified, with the novelty that the *Ayoteco* variety, which had been located taxonomically as *A*. *salmiana*, was revealed by molecular analyses to be more closely related to varieties of *A*. *mapisaga* [62]. In the State of Mexico, Alfaro-Rojas et al. [27] found six traditional varieties of pulque agaves, while in the state of Michoacán five have been recorded [21]. According to the previous comparative data, the State of Hidalgo is the state with the highest diversity of managed agaves, in addition to wild-growing agaves, from which some of the managed varieties and characteristics of the area have emerged, such as the traditional variety *Xaminí*.

Several authors agree that the genus *Agave* is highly variable, with high plasticity. This is due to the large number of ecotypes and clinotypes, as well as to the impact of factors such as arid environments and life history characteristics like pollination on *Agave* evolution [63–65]. In the case of pulque agaves, agrobiodiversity is due to various cultural motivations to keep these agaves in productive systems, and even though there is high agrobiodiversity in the Mexican highlands, in other localities we have noticed a trend of producers switching to crop systems with one or a few higher-yield varieties to increase production. There has also been an apparent increase in the intensity of management, with larger crop areas and increased use of agricultural technology such as the application of herbicides, though these trends need to be systematically corroborated.

### Management and domestication of pulque agave

In Mexico there is a wide diversity of systems and intensity of management of pulque agaves [66]. In this study, the management systems of *La Coyotera* and *El Cubo* corresponded to *Metepantles*, from nahuatl *metl*: agave, *pantli*: rows, a traditional agroforestry system that consists of rows of agave interspersed in plots of different plants, which maintain and conserve a high diversity of crops and native vegetation, such as *Corriente* or *Cimarron* agaves. The management of agaves has led to maintaining higher densities of traditional varieties that yield greater productivity in terms of quantity and quality of sap, as is the case of the *Manso de zoqui* variety. However, producers maintain and protect other varieties even when they are not so profitable, as part of their interest in safeguarding part of their cultural heritage. The continuous selection of some of these agave varieties has led to individuals presenting a larger rosette size and decreased thorniness, as has been recorded in other works exploring domestication syndrome in pulque and mezcal agaves [16,19–23,67]. However, for pulque agaves, the characteristics associated with the quantity and quality of their sap when it is edible have not been studied, as was noted in the domestication syndrome proposed by Colunga-García-Marín et al. [16]. Producers identified differences in the production and quality of the sap of the traditional varieties identified in this work (Table 2). Even so, it is pertinent to analyze in detail the daily and monthly sap production of the traditional varieties, as well as to quantify their sweetness, acidity, density, and color, among other organoleptic characteristics, and confirm the tendencies of domestication in some species.

It has been proposed that producers traditionally select vigorous, vegetatively propagated suckers, and that these plants can be polyploid, which are maintained for generations through clones [34,64]. Polyploidy is an important event in the evolution of angiosperms and provides several advantages over their diploid relatives. For example, polyploids have greater adaptability and responses to extreme environments, greater tolerance to cold, resistance to pathogens, and genotypic plasticity to use new habitats. About half of the species of the genus *Agave* are polyploid [68]; 40% in the subgenus *Littaea* and 64.3% of the subgenus *Agave* [64]. It is possible that the species recorded in this research exhibit various levels of ploidy. In *A*. *americana*, diploid (2x), tetraploid (4x), and hexaploid (6x) plants have been found. *A*. *mapisaga* presents sterile pentaploids (5x) [34,69], and in *A*. *salmiana* tetraploid and hexaploid plants (6x) have been recorded [34]. This may be one of the mechanisms of gigantism in these species.

For future analyzes of agave populations, we consider it important to include, in addition to the taxonomic classification, the classification of traditional varieties used by producers in each specific region, since this classification system allows analyzing the artificial selection processes to which agave populations are subject, both domesticated species in crops, and wild species that are being managed.

### Genetic diversity and structure of pulque agaves

For the varieties and species in this study, the low levels of observed heterozygosity compared to expected heterozygosity and the high levels of inbreeding may be related to the presence of null alleles with frequencies greater than 8%, which may increase the parameters of population differentiation such as F_ST_, F_IS_ and decrease genetic diversity [53]. On the other hand, the presence of null alleles may be due to the vegetative propagation of the same genotype in crops. It may also be due to the fact that we assumed the individuals to be diploid, even though polyploids are found in these species. It is therefore possible that we have underestimated the allelic richness and the presence of heterozygous individuals, identifying false homozygotes.

According to Eguiarte et al. [70], agaves exhibit similar levels of genetic diversity and population differentiation (H_S_ = 0.190, F_ST_ = 0.150) to monocot angiosperms in isoenzymes (H_ES_ = 0.158, G_ST_ = 0.157; [71]) and RAPDs (H_POP_ = 0.190, G_ST_ = 0.31; [72]). The diversity and genetic structure values we found were higher than those reported in those works, for the traditional varieties of pulque agave, the management categories, and the taxonomic identities. However, the molecular markers used in these studies were low-polymorphism markers, so comparisons must be made with care. On the other hand, Álvarez-Ríos et al. [21] evaluated the genetic diversity of five varieties of pulque agaves in *linderos* management systems in Michoacán using nuclear microsatellites and found similar levels of species-level genetic diversity (He) to our values, although their values for the species *A*. *americana* were higher than ours ([21]; He = 0.527 vs this study; He = 0.290)].

The hypothesis of higher genetic diversity in wild than cultivated populations of pulque agaves has been supported by several authors previously. Figueredo et al. [20] found that the cultivated species *A*. *hookeri* (He = 0.485) presented lower levels of genetic diversity than its wild ancestor, *A*. *inaequidens* (He = 0.704). Similarly, Alfaro-Rojas et al. [27] found that genetic diversity was low for the cultivated species *A*. *salmiana* var. *salmiana* (*Manso*, H = 0.121; *Ayoteco*, H = 0.119) and *A*. *mapisaga* (*Carrizo*, H = 0.086) in northeastern Mexico state using RAPD’s, which compared to the high levels of diversity recorded in wild populations of *A*. *salmiana* ssp. *crassispina* in San Luis Potosí, using AFLP’s (Hs = 0.408). The low genetic diversity in pulque agaves may be due to intrinsic characteristics of the plant, as well as the fact that the crops have mostly been propagated vegetatively, possibly for the past few thousand years, according to archaeological evidence. In addition, the fact that the management of plants eliminates the possibility of sexual reproduction, also prevents genetic recombination.

In the case of agaves used to produce traditional mescal, patterns of genetic diversity have been found to be the opposite of those reported for pulque agaves. This high diversity among cultivated varieties is due to the fact that there is a wide range of management systems, where traditional agroforestry systems (TAFS) are a reservoir for a high number of traditional varieties, morphological and genetic diversity, due to the frequent and constant introduction of plants from other sources in these systems, effective management practices such as the formation of seedbeds and the implementation of management plans to promote and maintain sexual reproduction. In addition, there is historical information that in some species of agaves the production of mezcal began approximately 400 years ago, with cultivation being an even more recent phenomenon (approximately 30 years) [20].

## Conclusions

We found a high agrobiodiversity of agaves, which are maintained in traditional agroforestry systems such as *Metepantles* and backyard gardens. The varieties studied presented a great diversity in their form, which is a product of the varied environments where these agaves grow as well as the diverse cultural motivations of pulque producers to maintain diversity in their crops and management practices. As such, these systems should be considered productive spaces that maintain diversity, practices that should be maintained in other productive systems in the country. The species with the greatest diversity of forms and traditional varieties was *Agave salmiana*, while the species that most clearly exhibited the domestication syndrome was *A*. *mapisaga*. The wild-collected agave species showed high levels of genetic diversity, while the cultivated varieties exhibited low levels of diversity, probably due to vegetative propagation. Varieties such as *Corrientes* or *Cimarrones* (*A*. *salmiana* ssp. *crassispina*) are found in the natural ecosystems, which eventually enrich the gene pool of the crops through their occasional introduction into crops (Tolerated and Transplanted). Similarly, some cultivated agaves "escape" (reproduce sexually), which in turn has maintained some diversity in the agave populations.

## Supporting information

S1 File Interview on the use and management of pulque agave in Hidalgo. (DOCX)
