# Supplementary material for: Morphological and genetic diversity of traditional varieties of agave in Hidalgo State, Mexico
Source: PLoS One. 2021 Jul 9;16(7):e0254376. doi: 10.1371/journal.pone.0254376 (PMC8270473; doi:10.1371/journal.pone.0254376)
Supplement: S1 File — (DOCX) [file pone.0254376.s001.docx]

s1 File. INTERVIEW ON THE USE AND MANAGEMENT OF Pulque AGAVE IN HIDALGO

Location:

Date:

Interviewee name:

Gender:

Age:

Occupation:

I. AGAVE MANAGEMENT

What parts of the plant do you use?

a) root b) leaf c) stem (corm) d) green floral stalk e) dry floral stalk f) flowers g) fruits h) whole plant.

How long have you been using / working with agaves?

Where do grow the agaves you use (size and brief description of the structure of the space)?

What type of property is your field? (ejidal, communal, private property, rented plot)

Where do the agaves you have/handle come from? Are they from here, or were they brought from elsewhere?

If you bought the agaves, where did you buy them?

Are your agaves …

a) planted, b) wild growing, or c) both

If you sow, what material do you use?

a) seeds b) bulbils c) seedlings d) a mixture

What are your practices for planting agaves?

What practices do you use to care for or keep your agaves in good condition?

What are the practices you use to propagate or keep agaves abundant?

How many agaves do you have? Aproximately?

Do you grow any other plants or raise animals?

II. MORPHOLOGICAL DIFFERENCES OF AGAVES (IDENTIFY AND DESCRIBE VARIETIES)

1. How many varieties (types) of pulque agave do you know?

2 Which of these varieties of agaves do you have in your fields?

3. Describe each of the varieties (size, color, suckers, fast or slow growth, other characteristics)

4. Are there varieties of agave in surrounding ecosystems? For example, in the *cerro* (the hill, xerophilous scrub) or *monte* (the montains, pine-oak forest)?

5. Describe each of the varieties of agave in the *cerro* (the hill, xerophilous srub) or *monte* (the montains, pine-oak forest).

6. Which variety do you prefer? Why?

a) larger b) fewer thorns c) produce higher quality sap d) more drought resistant e) have other uses

7. What similarities are there between cultivated agaves and those of the *cerro* (the hill, xerophilous scrub) or *monte* (the mountains, pine-oak forest)?

8. What differences are there between cultivated agaves and those of the *cerro* (the hill, xerophilous scrub) or *monte* (the mountains, pine-oak forest)?

9. What are the most important characteristics for identifying an agave?

*If not previously described in sufficient detail, ask the following questions with an emphasis on the characteristics:*

10. What color are the agave varieties you handle? (to lean on ranking from darker to lighter)

11. How would you describe the spines of the varieties you handle? (to lean on ranking from large to small)

12. How large is the plant of the varieties you handle? (to lean on ranking from large to small)

13. What is the size of the leaves of the varieties you handle? (to lean on ranking from large to small).

III. PHYSIOLOGICAL DIFFERENCES AMONG VARIETIES (SAPONINS, SUGARS)

1. Are there varieties of agaves that when touched or handled cause *roña* (itching, i.e., contact dermatitis) Which varieties?

2. Do those agaves grow in cultivated fields or in the surrounding ecosystems?

3. Do some varieties have sweeter sap than others? Which? (to lean on ranking from sweetest to least sweet)

4. Do those plants grow in cultivated fields or in the surrounding ecosystem?

5. Are there varieties that produce more sap in one day than others? Which? (lean on ranking from higher to lower daily sap production)

6. Do those plants grow in cultivated fields or in the surrounding ecosystem?

7. Are there varieties that produce more sap for a longer time (months) than others? Which? (to lean on ranking from highest to lowest production duration).

8. Do those plants grow in cultivated fields or in the surrounding ecosystem?

IV. PULQUE PRODUCTION

1. How long have you been producing pulque?

2. Who taught you to produce pulque?

a) parents b) grandparents c) uncles d) friends e) other (specify)

3. What agaves do you prefer for producing Pulque and why?

a) the largest b) the smallest c) those with more leaves d) those with fewer spines e) the most accessible f) those in the sun g) those in the shade h) those that appear healthy i) those that cause less itching (contact dermatitis) j) any k) other (specify)

4. Where are these plants found?

5. When do you produce pulque?

6. Does anyone help you in production? If so, at what stage do they help? (sowing agaves, harvesting sap, making pulque, etc.)

7. How many agaves do you have in production simultaneously?

8. How much sap do you extract (total) per day?

9. What do you do with your daily production? Specify percentage for each:

a) self-consumption b) sale c) other (specify)

10. Where do you sell your sap/pulque?

11. How much do you sell it for? (sap and pulque)

12. What do pulque agave and pulque mean to you?
